# Extrachromosomal circular DNA are functional, heritable units that expand genomic plasticity and confer resilience

**DOI:** 10.3389/fpls.2026.1770110

**Published:** 2026-02-25

**Authors:** Dana R. MacGregor, Christopher A. Saski

**Affiliations:** 1 Rothamsted Research, Harpenden, Hertfordshire, United Kingdom; 2 Department of Plant and Environmental Sciences, Clemson University, Clemson, SC, United States

**Keywords:** extrachromosomal circular DNA (eccDNA), functional genomics, non-Mendelian inheritance, phenotypic plasticity, rapid adaptation, stress tolerance

## Abstract

Although far less well-known and understood than chromosomal DNA, extrachromosomal circular DNA (eccDNA) are a pervasive and dynamic component of eukaryotic genomes. eccDNA are nuclear-localized, double-stranded DNA circles that exist independently of the main chromatin body. They share many sequence features with chromosomal DNA, including encoding functional genes; however, unlike chromosomes, eccDNAs are highly heterogenous, capable of autonomous replication and ultra-high gene expression, and do not necessarily segregate evenly or follow Mendelian inheritance during cell division. Although several recent reviews have focused on their roles in human health, emerging research in plants shows that eccDNAs are intricately associated with rapid adaptation to stress, particularly in weedy and invasive plants. This plant-centric review synthesizes evidence that eccDNAs carry full-length genes, regulatory elements, and transposable sequences, that collectively enable gene amplification, novel protein variants, and context-specific expression. We propose that eccDNAs function as “genomic shock absorbers”: stress-inducible, non-Mendelian reservoirs of genetic diversity that buffer genomes against environmental challenges such as nutrient limitation and xenobiotic exposure. Drawing parallels with bacterial plasmids, we argue that eccDNA facilitate novel and important genome–environment interactions beyond those mediated by chromosomes. Harnessing these elements as non-Mendelian vehicles for genetic innovation could offer a route to translate weed-derived resilience into novel crop improvement strategies, enabling the design of climate-ready, stress-resilient agriculture grounded in weed inspired mechanisms of adaptability and tolerance.

## 1 Introduction

The discovery and renewed investigation of extrachromosomal circular DNA (eccDNA) are redefining the concept of “heritable genetic material”. eccDNAs are defined as double-stranded circular DNA molecules that exist independently from chromosomal DNA. eccDNA have been found in classical model organisms like yeast (Møller et al., 2015b), Drosophila (Stanfield and Helinski, 1976), frogs (Cohen and Segal, 2009) and mice (Alt et al., 1978), phytopathogens like *Magnaporthe oryzae* (Joubert and Krasileva, 2022), as well as from a variety of non-model organisms like boar (Hotta and Bassel, 1965) and pigeons (Møller et al., 2020). They have also been widely reported from human samples (e.g. Kumar et al. (2017) and reviewed in Ling et al. (2021)). Most relevantly here, eccDNA are present in a diverse range of plants (**Table 1**). The population of eccDNAs within a sample – its “circulome” – is highly heterogenous in size, abundance, and sequence content, and dynamically varies across cells and conditions. Functioning as an active complement to the relatively fixed chromosomal genome, the circulome can reshape genetic architecture, influence phenotypic outcomes, and represents a functional extension of Crick’s “central dogma of molecular biology” (Crick, 1970). Throughout this review, we use “eccDNA” as an umbrella term for nuclear-localized extrachromosomal DNA circles, spanning small gene-sized circles (also referred to as “microDNA” or “spcDNA” in other systems) through to larger eccDNA-like replicons that can reach hundreds of kilobases. We explicitly distinguish these nuclear eccDNAs from (i) organellar circular genomes/sub-genomic circles such as chloroplast and mitochondrial DNA and from (ii) oncology “ecDNA” or “double minutes”, which typically denotes large oncogene-amplifying circles in cancer. When drawing analogies to cancer biology, we use “ecDNA” in that field-specific sense for mechanistic comparison only.

**Table 1:** Overview of reported eccDNA across plant species.

Authors, Year & DOI	Species	eccDNA class	Mechanism	Phenotype
Kinoshita et al. (1985) DOI: 10.1093/oxfordjournals.pcp.a077040	*Triticum aestivum* (wheat); *Nicotiana tabacum* (tobacco)	spcDNA/eccDNA (nuclear fraction)	Nuclear origin; extrachromosomal circles (mechanism not resolved)	None (descriptive localization/structure)
Cohen et al. (2008) DOI: 10.1111/j.1365-313X.2007.03394.x	Multiple higher-plant species (survey across small & large genomes)	Tandem-repeat eccDNA (satellite/centromeric repeats)	Homologous recombination; tandem-repeat excision; repeat array dynamics	None (proposed role in genome plasticity)
Navrátilová et al. (2008) DOI: 10.1186/1471-2229-8-90	Ten higher-plant species incl. *Arabidopsis, Oryza, Pisum, Secale, Triticum, Vicia*	Satellite repeat–derived eccDNA (nicked circles; multimers)	Homologous recombination; long sequence similarity; possible rolling-circle amplification models	None (implicated in satellite evolution)
Lanciano et al. (2017) DOI: 10.1371/journal.pgen.1006630	*Arabidopsis thaliana; Oryza sativa* (rice)	TE-derived eccDNA (LTR retrotransposons; mobilome)	Circularization of extrachromosomal TE DNA; NHEJ/HR; TE life cycle intermediates	None (activity marker); identifies active retrotransposons (e.g., EVD, Tos17, PopRice)
Cho et al. (2019) DOI: 10.1038/s41477-018-0320-9	*Oryza sativa* (rice) (ALE-seq demonstrated in crops)	Extrachromosomal circular/linear DNA intermediates of LTR retrotransposons (pre-integration)	LTR amplification from ecl/eccDNA; retrotransposition intermediates; stress-induced TE activation	None (method enabling detection of active LTR-RTs)
Benoit et al. (2019) DOI: 10.1371/journal.pgen.1008370	*Solanum lycopersicum* (tomato)	LTR retrotransposon eccDNA (Rider)	LTR retrotransposition intermediates; ABA-responsive activation; epigenetic silencing (siRNA/RdDM)	Potential contribution to genetic/epigenetic variation (TE mobilization)
Esposito et al. (2019) DOI: 10.1007/s00425-019-03283-3	*Solanum tuberosum* (cultivated potato); *Solanum commersonii* (wild potato relative)	LTR retrotransposon-associated eccDNA (mobilome-seq marker of activity; ‘nightshade’ element)	Circularization by host DNA repair; stress-associated TE activity	None (TE activity characterization)
Mehta et al. (2020) DOI: 10.1038/s41596-020-0301-0	*Arabidopsis thaliana* (method demonstrated; also circular DNA viruses)	Method paper (CIDER-Seq for full-length circular DNA incl. eccDNA)	Rolling-circle amplification deconcatenation; full-length circular molecule reconstruction	Not applicable
Molin et al. (2020c) DOI: 10.1105/tpc.20.00099	*Amaranthus palmeri* (Palmer amaranth)	Gene-bearing eccDNA replicon (EPSPS amplification; large eccDNA/ecDNA-like)	Autonomous replication; tethering/segregation; recombination-mediated formation; amplification	Glyphosate resistance via EPSPS gene amplification on eccDNA
Molin et al. (2020b) DOI: 10.1186/s13104-020-05169-0	*Amaranthus palmeri* eccDNA tested in *Saccharomyces cerevisiae* (functional assay)	Replication origin element (ARS) within A. palmeri eccDNA replicon	Replication origin activity; autonomous maintenance	Supports autonomous replication capability (mechanistic evidence)
Wang et al. (2021) DOI: 10.1016/j.csbj.2021.01.043	*Arabidopsis thaliana* (multiple tissues)	Genome-wide eccDNA (genic, intergenic, TE-derived)	Direct repeats at junctions; DSB repair signatures; TE activity contribution	Tissue-specific eccDNA landscapes (functional implications largely unresolved)
Kwolek et al. (2022) DOI: 10.1111/tpj.15773	*Daucus carota* (carrot)	LTR retrotransposon eccDNA (DcAle lineage; Alex1/Alex3)	TE activation in culture; eccDNA as marker of recent activity	*De novo* insertions documented; genome diversity in callus
Koo et al. (2018) DOI: 10.1073/pnas.1719354115	*Amaranthus palmeri* (Palmer amaranth)	Gene-bearing eccDNA replicon (EPSPS amplification)	Extrachromosomal replication; non-Mendelian inheritance; replication/segregation dynamics	Glyphosate resistance
Spier Camposano et al. (2022) DOI: 10.1371/journal.pone.0260906	*Amaranthus palmeri* (Palmer amaranth)	Population eccDNA diversity (incl. small circles; EPSPS-associated structures)	Structural heterogeneity; excision/amplification variation	Links eccDNA landscape to glyphosate resistance context
Fu et al. (2023) DOI: 10.3390/genes14101905	*Alopecurus myosuroides* (blackgrass)	Gene-bearing eccDNA (GSTF1-containing; metabolic resistance-associated)	Gene amplification on eccDNA; possible autonomous replication; structural maintenance	Herbicide resistance (metabolic; GST-based)
Koo et al. (2023) DOI: 10.1093/plphys/kiad281	*Amaranthus palmeri; Amaranthus tuberculatus* (waterhemp)	Transmissible eccDNA replicon (EPSPS) via hybridization	Interspecific transmission; maintenance/replication; non-Mendelian segregation	Spread of glyphosate resistance across species
Merkulov et al. (2023) DOI: 10.3390/plants12112178	*Arabidopsis thaliana*	TE-derived eccDNA (ONSEN/EVD; full-length vs truncated)	TE-derived eccDNA structure; stress-dependent eccDNA forms; truncation patterns	None (structural/quantitative eccDNA response to stress)
Zhang et al. (2023) DOI: 10.1038/s41467-023-41023-0	*Arabidopsis thaliana*	TE-eccDNA + structural variants in epigenetic mutants	Epigenetic control; TE eccDNA load; genome instability; SV formation	Genome instability signatures; TE insertions
Zhuang et al. (2024) DOI: 10.1038/s41467-024-46691-0	*Oryza sativa* (rice)	Genome-wide eccDNA (tissue-specific; single- and multi-fragment circles)	Direct repeats at junctions; random formation; minimal gene-expression impact reported	None (landscape + formation rules)
Zhang et al. (2024) DOI: 10.1186/s43897-024-00124-0	*Solanaceae* graft system (Lycium ‘goji’ stock → *Solanum lycopersicum* scion; regenerants)	Plasmid-like eccDNAs transferred across graft junctions	Horizontal transfer; plasmid-like eccDNA exchange; replication/maintenance in regenerants	Pleiotropic traits; perennial-biased growth and agronomic changes in ‘Go-tomato’
Merkulov et al. (2024) DOI: 10.1186/s12864-024-10314-1	*Solanum lycopersicum* (breeding line) with *S. peruvianum* introgressions	TE-derived eccDNA (tomato LTR retrotransposons ‘Salsa’ and ‘Ketchup’)	LTR retrotransposition intermediates; introgressed TE activity; eccDNA structure	Potential source of breeding-line genetic/phenotypic diversity (TE activity)
Ni et al. (2025) DOI: 10.1038/s41467-025-59572-x	*Oryza sativa* (rice)	Single- and multiple-fragment eccDNA under nutrient stress	Adaptive dynamics; stress-associated eccDNA formation; repeat-mediated junctions	Stress-response–associated eccDNA landscape changes (functional links explored)
Zaidi et al. (2025) DOI: 10.1371/journal.pbio.3003275	*Arabidopsis thaliana*	Full-length eccDNA atlas (TE-derived hotspots; long-read)	Epigenetic regulation of TE-eccDNA; centromeric/pericentromeric hotspots	Resource/atlas (functional implications: TE regulation)
Carvalho-Moore et al. (2025) DOI: 10.1093/plcell/koaf069	*Amaranthus palmeri*	Rearranged multi-gene eccDNA replicon (EPSPS + GS2)	eccDNA rearrangement; co-duplication; autonomous replication; structural variation	Dual herbicide resistance (glyphosate and glufosinate)
Merkulov et al. (2026) DOI: 10.3390/ijms27010286	*Nicotiana benthamiana; Arabidopsis thaliana; Brassica napus*	Virus- and stress-associated TE eccDNA (Galadriel Ty3-retrotransposons in N. benthamiana)	Viral suppressor of RNA silencing; HSF motifs; TE de-repression; eccDNA generation	Genome/mobilome changes associated with viral infection and heat stress

Several dedicated eccDNA databases (e.g. Guo et al. (2023); Zhong et al. (2023); Ni et al. (2025)) have begun to consolidate and organize circulome datasets. However, plants in general remain underrepresented in these despite the early discovery of eccDNA in wheat (Hotta and Bassel, 1965) and plant-focused reviews that have highlighted the existing plant-focused literature. For instance, Peng et al. (2022) positioned eccDNA as a neglected but potentially adaptive nucleic-acid pool in plants and highlighted core approaches for circulome profiling. More recent reviews have reinforced these themes, with Mohan et al. (2024) explicitly discussing eccDNA as a future biotechnological tool for plant stress resilience and Azam et al. (2024) providing a broader, technology-oriented perspective. Here, we synthesize evidence from plants, particularly from weedy and invasive species, showing that eccDNAs can carry functional coding/regulatory content and contribute to stress adaptation via copy-number and regulatory shifts. We then summarize biogenesis/inheritance mechanisms and evaluate how weed circulomes might inform crop-improvement concepts.

## 2 eccDNA as drivers of genome plasticity and environmental response

**Figure 1:**
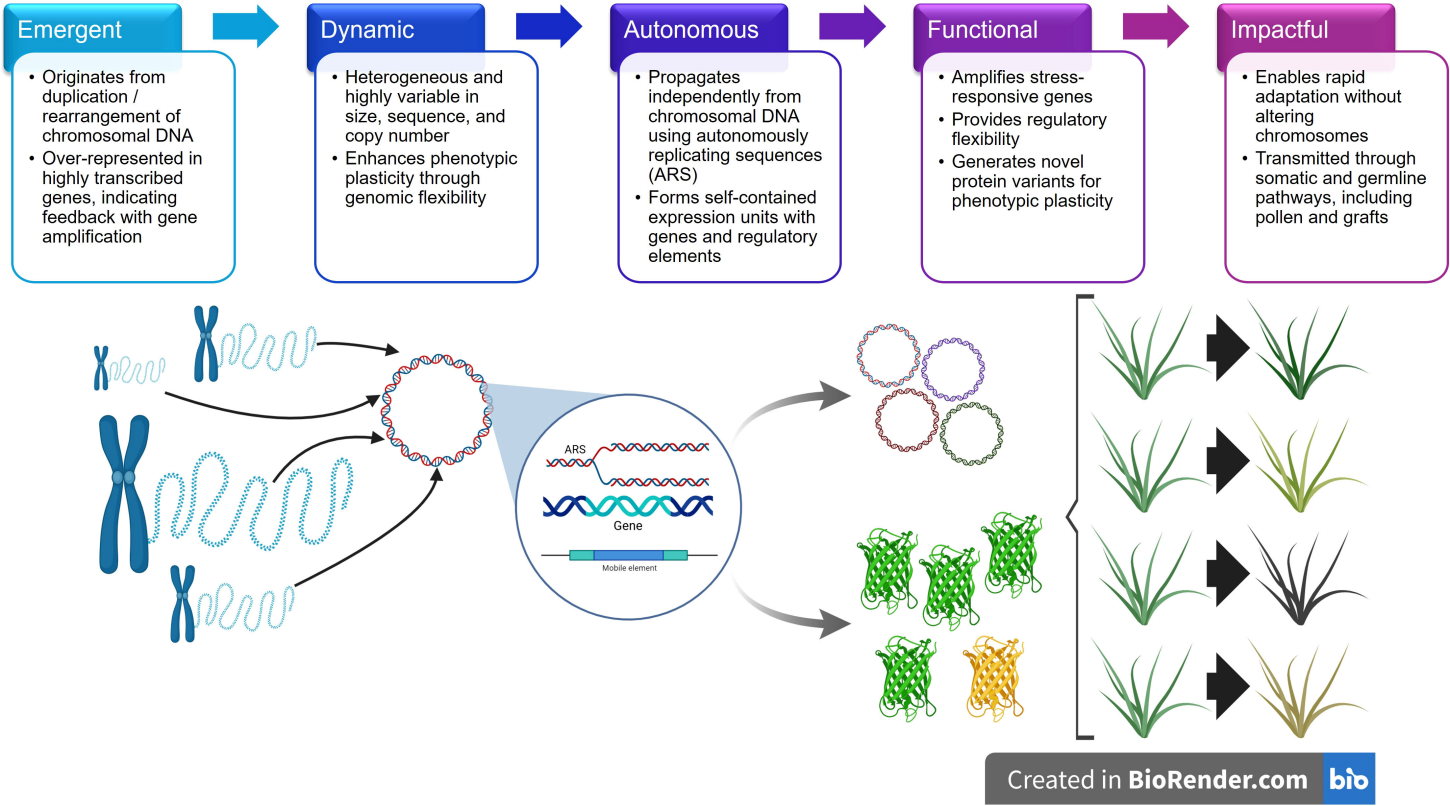
Extrachromosomal circular DNA (eccDNA) in plants exhibits five key properties that collectively enable rapid genomic innovation and phenotypic flexibility. Dynamic: Highly variable in size, sequence, and copy number, enhancing phenotypic plasticity through genomic flexibility. Functional: Amplifies stress-responsive genes, provides regulatory flexibility, and generates novel protein variants. Impactful: Enables rapid adaptation without altering chromosomes and is transmitted through somatic and germline pathways, including pollen and grafts. Emergent: Originates from duplication and rearrangement of chromosomal DNA and over-represents highly transcribed genes, indicating feedback with gene amplification. Autonomous: Propagates independently using autonomously replicating sequences (ARS) and forms self-contained expression units with genes and regulatory elements. Together, these properties position eccDNA as a native, modular system for non-Mendelian inheritance and stress-responsive adaptation in plants.

Recognizing eccDNA as a functional layer of genomic plasticity reframes how we understand heritability, variability, and adaptability in plants. These concepts are synthesized in **Figure 1**, which illustrates five defining properties of plant eccDNA – Emergent, Dynamic, Autonomous, Functional, and Impactful – and shows how they collectively position eccDNA as a plasmid-like system for adaptive genome plasticity. Ultimately, elucidating the mechanisms that govern eccDNA formation, inheritance, and function may open new avenues for non-GMO crop improvement and provide a blueprint for engineering the adaptability that defines successful weeds into climate-resilient, future-proofed crops.

Evolution is traditionally conceived as a gradual process governed by mutation and selection acting on chromosomal DNA, yet this paradigm is incomplete. From Charles Darwin’s recognition that genetic variation fuels selection (Darwin, 1859) to Barbara McClintock’s vision of genomes as responsive systems capable of “sensing shocks” and “mitigating danger” (Mcclintock, 1984), it is clear that genomes possess intrinsic mechanisms to accelerate adaptation under stress. Emerging insights into the role of the circulome in rapid adaptation call for a broader view of genome dynamics, one in which eccDNA generation, propagation, and functionalization emerge as central mechanisms for within-generation, stress-responsive adaptation.

### 2.1 Darwinian outcomes through non-Mendelian routes

A variety of theories regarding eccDNA biogenesis have been proposed and are well covered elsewhere (e.g (Gaubatz, 1990; Kuttler and Mai, 2007; Møller et al., 2015a; Cao et al., 2021; Ling et al., 2021; Li et al., 2022; Yao et al., 2025). Most suggest that chromosomal DNA is copied and pasted into eccDNA through processes involving transposable elements (Kwolek et al., 2022; Zaidi et al., 2025), direct repeats (Zhuang et al., 2024), or the repair of DNA double-strand breaks (Vanloon et al., 1994). Current data indicate that there is an element of chance in which chromosomal segments are transferred, as eccDNA sequences exhibit “shotgun coverage” across the genome (Spier Camposano et al., 2022; Fu et al., 2023; Ni et al., 2025). Additionally, the data suggest that eccDNA can also arise through coordinated mobilization from specific chromosomal loci (Kuttler and Mai, 2007; Song et al., 2025; Zaidi et al., 2025). For example, comparative genomics among *Amaranthus* species indicates that higher order chromatin interactions influence the transfer of sequences from chromosomes to eccDNA (Molin et al., 2020c). Even though substantially more research is required before the mechanism(s) that mediate DNA movement into eccDNA are fully understood, the current evidence suggests that both random and regulated processes contribute to their formation and maintenance.

Although how and which DNA gets into an eccDNA is not well understood, it is clear that the number of eccDNAs increase and novel sequence content appears within the circulome in response to developmental cues or environmental stressors such as xenobiotic exposure, pathogen attack, disease, or aging; these references are summarized in **Table 2**. Many eccDNAs possess autonomous replication sequences (Møller et al., 2015b; Molin et al., 2020b), and segregate unevenly during meiotic and mitotic cell divisions (Koo et al., 2018; Ni et al., 2025), further amplifying variability. Recent theoretical work is beginning to formalize these dynamics in quantitative terms. Arrey et al. (2022) propose a unifying five-factor framework: 1) formation, 2) replication, 3) segregation, 4) selection, and 5) elimination to explain how cellular eccDNA “load” changes over time, explicitly capturing how uneven segregation decouples eccDNA inheritance from strict 1:1 Mendelian expectations. This sequential interplay of factors in this model explains temporal changes in eccDNA abundance, as initial formation under stress can lead to replication-driven amplification, followed by biased segregation that amplifies variability, selective retention of adaptive variants, and eventual elimination once pressures subside, thereby facilitating non-Mendelian genetic plasticity. Applying such models to plant circulomes could enable predictive tracking of eccDNA-driven adaptation; for instance, in weeds evolving across herbicide gradients, simulations might forecast when resistance-conferring eccDNA amplifications (e.g., *EPSPS* replicons in *Amaranthus palmeri*) arise and persist, or in crops under cyclic heat/drought, predict the emergence and collapse of resilience traits, informing breeding strategies for stress-tolerant varieties by identifying intervention points to harness or mitigate these dynamics.

**Table 2:** Summary of studies reporting changes in the abundance, composition, formation, or functional significance of eccDNA across organisms in response to developmental cues or environmental stressors.

Authors and year	Study type/methods	Organism/system/context	Key findings	Limitations/caveats	Major impact
Stanfield and Helinski (1976) DOI:10.1016/0092-8674(76)90123-9	Electron microscopy; density centrifugation; restriction digestion; drug perturbation (cycloheximide/puromycin)	*Drosophila melanogaster* embryos and Schneider line 2 cells; protein synthesis inhibitor treatment	Nuclear small circular DNA is present and increases ~30-fold after 14 h cycloheximide/puromycin; circles are size-heterogeneous	No sequence-level resolution; early methods; functional roles not established	Evidence that xenobiotic stressors can rapidly increase eccDNA abundance in eukaryotic cells
Thieme et al. (2017) DOI: 10.1186/s13059-017-1265-4	Inhibition of RNA polymerase II (Pol II); stress assays; mobilome sequencing; extrachromosomal retrotransposon DNA detection	*Arabidopsis thaliana* and *Oryza sativa* under stress and Pol II inhibition	Inhibition of DNA methylation and Pol II increases production of extrachromosomal retrotransposon DNA, enabling strong heat-dependent mobilization of ONSEN with up to 75 new heritable insertions	Mechanistic links not fully resolved; focus on ONSEN retrotransposon	Demonstrates activation of retrotransposons via Pol II and DNA methylation inhibition,
Koo et al. (2018) DOI:10.1073/pnas.1719354115	Fluorescence *in situ* hybridization (FISH); cytogenetics; inheritance studies	*Amaranthus palmeri*; glyphosate resistance	Copies of *EPSPS* reside on eccDNA (‘eccDNA replicon’) that anchors to chromosomes, segregates in non-Mendelian fashion, and confers herbicide resistance	Molecular origins unresolved at the time; limited mechanistic detail	Direct link between xenobiotic (glyphosate) exposure and eccDNA-mediated adaptation in weeds
Esposito et al. (2019) DOI:10.1007/s00425-019-03283-3	TE activity profiling; cold-stress induction; mobility assays	*Solanum tuberosum* and *S. commersonii* under cold stress	Cold stress in leaves activates LTR retrotransposon, eccDNA intermediates and mobility	Focus on TE activity; eccDNA not fully structurally resolved	Adds evidence for stress-induced TE-eccDNA dynamics in crops
Møller et al. (2020) DOI:10.1093/gbe/evz281	eccDNA enrichment and sequencing across tissues; comparative analysis	Different breeds of domestic pigeon (*Columba livia domestica*) differing in flight behavior vs human skeletal muscle/blood	Tens of thousands of eccDNAs detected; nonflying pigeons show higher counts than homing pigeons; high numbers of unique eccDNA types per human nucleus	Observational; causality with physiology not tested	Supports physiological or state-dependent variation in circulome size across breeds and species
Joubert and Krasileva (2022) DOI:10.1186/s12915-022-01457-2	Whole-circulome sequencing; comparative genomics	Fungal pathogen *Magnaporthe oryzae*	Diverse eccDNAs enriched for effectors and genes in presence–absence variable regions; disease-associated genes frequently on eccDNAs	Functional consequences mostly inferred	Links pathogen pressure/disease context to dynamic circulome content
Spier Camposano et al. (2022) DOI:10.1371/journal.pone.0260906	CIDER-Seq long-read circular DNA profiling; comparative analysis	*Amaranthus palmeri*; glyphosate-resistant vs -sensitive biotypes	Thousands of eccDNAs; GR biotypes possess abundant eccDNA fragments that assemble into a near-complete EPSPS replicon; hotspots for eccDNA formation	Population sizes modest; functional tests limited	Demonstrates xenobiotic-associated expansion and assembly of circulome elements
Fu et al. (2023) DOI:10.3390/genes14101905	CIDER-Seq profiling; genome mapping; domain annotation	*Alopecurus myosuroides* (herbicide-resistant and -sensitive populations)	eccDNAs present across HR and HS populations; detoxification genes (P450s, ABC transporters, GSTs incl. *AmGSTF1*) on eccDNA; genomic hotspots overlap resistance QTLs	Association not causation; functional validation needed	Direct evidence in blackgrass linking circulome content to resistance-associated loci
Koo et al. (2023) DOI:10.1093/plphys/kiad281	Experimental hybridization; FISH mapping	*Amaranthus* interspecific hybrids (*A. palmeri, A. tuberculatus, A.* sp*inosus*)	Pollen-mediated transfer of the EPSPS eccDNA replicon across species; random anchoring; massive somatic copy-number variation	Focus on EPSPS replicon; broader gene content not dissected	Highlights gene-flow risk for eccDNA-mediated resistance across weed species
Zhuang et al. (2024) DOI:10.1038/s41467-024-46691-0	Circle-Seq across tissues; motif and deletion analyses	*Oryza sativa*; six tissues; environmental light stress assays	Identified 25,598 eccDNAs across tissues; direct repeats implicated in formation; leaf-specific accumulation; association with minor chromosomal deletions; increased eccDNA under intense light	Limited functional assays on gene expression impact	Demonstrates developmental tissue differences and environmental (light) stress effects on plant circulome
Gu et al. (2025) DOI:10.1371/journal.pone.0324438	Computational meta-analysis across cancers and health; algorithm development (eccDriver)	Human	Catalogued eccDNA-driven genes (EDGs); 27 common EDGs and a 17-gene prognostic signature; 432 candidate drivers, many druggable	Human-focused; relies on public datasets; circular causality not proven	Analytical framework for defining eccDNA-driven genes
Ni et al. (2025) DOI:10.1038/s41467-025-59572-x	eccDNA profiling across development; nutrient (N/P) deficiency treatments; classification of ecGenes/ecTEs; mechanistic inference	*Oryza sativa* under nitrogen and phosphorus deficiency	eccDNA landscape shifts with development and nutrient stress; TE-related eccDNAs increase under P/N stress; proposed TE-mediated homologous recombination for multi-fragment eccDNAs	Primarily associative; limited functional perturbations	Shows abiotic stress (nutrient) reshapes plant circulome content and class composition
Song et al. (2025) DOI: https://doi.org/10.1111/pce.15549	Circle-Seq; RNA-Seq integration; motif analysis	*Pinus tabuliformis* vascular cambium across annual cycle	eccDNAs present throughout cambium cycle with exon preference and AA/AT/TT/TA junction repeats; strongest dynamic changes during dormancy	Woody species; functional validation needed	Demonstrates developmental cue–linked shifts in circulome in a perennial plant
Merkulov et al. (2026) DOI: 10.3390/ijms27010286	Virus infection assays; transcriptomics; eccDNA detection	*Arabidopsis thaliana*, *Brassica napus*, and *Nicotiana benthamiana* infected with Tobacco rattle virus (TRV), Potato virus X (PVX), and Tobacco ringspot virus (TRSV).	Viral infection activates LTR retrotransposon transcription and triggers eccDNA formation	Mechanistic links between virus-induced TE activation and eccDNA generation require further study	Demonstrates biotic stress (virus) as inducer of TE-eccDNA and genome plasticity

The dynamism of eccDNA extends beyond individual organisms. There is evidence that specific eccDNA variants can be transferred between species via pollen (Koo et al., 2023) or across graft junctions (Zhang et al., 2024). So far, interspecific transfer of eccDNA has only been documented in plants, perhaps because experimental approaches that directly test for horizontal DNA movement are more readily implemented in plants than in other taxa. Taken together with evidence linking eccDNA to gain-of-function phenotypes (Alt et al., 1978; Shoura et al., 2017; Hull et al., 2019; Molin et al., 2020c; Fu et al., 2023; Koo et al., 2023; Zhang et al., 2024; Ni et al., 2025; Zaidi et al., 2025), these observations suggest that eccDNA variants that confer an adaptive advantage can be propagated, distributed, and inherited. Consequently, eccDNAs represent a mechanism through which genetic novelty and advantageous traits can spread, positioning them as heritable genetic material that contributes to Darwinian evolution vial routes that are not traditionally Mendelian.

Despite this mounting evidence for high heterogeneity among eccDNAs and circulomes, patterns of abundance and composition across developmental stages and stress conditions indicate certain eccDNA sequences are consistently enriched in circulome samples (Wang et al., 2021; Chamorro González et al., 2023; Fu et al., 2023). One striking example is the “eccDNA Replicon”, an intricate arrangement of repetitive sequences and mobile genetic elements interspersed with clusters of palindromic arrays thought to support stability, tethering, and potentially nuclear integration of adjacent or intervening sequences (Molin et al., 2020c). Importantly, this eccDNA Replicon was shown to carry multiple copies of the *5-Enolpyruvylshikimate-3-phosphate synthase* (*EPSPS*) gene, and analyses of this eccDNA Replicon provided the first documented example where an eccDNA was functionally validated to confer glyphosate resistance in plants (Molin et al., 2017; Koo et al., 2018; Molin et al., 2020a, 2020). The eccDNA Replicon is unusually large (~400 kb) and is composed of sequences, including additional genes, originating from distal regions of the genome, which are likely to have been assembled through recombination among smaller eccDNAs and shaped by selection under environmental pressures over a short evolutionary timescale. Interestingly, the eccDNA Replicon was recently found to have incorporated the coding sequence for Glutamine Synthetase 2 (GS2) into its amplicon, conferring glufosinate resistance and further supporting the idea of recombination and “internal crosstalk” among eccDNAs within the circulome (Carvalho-Moore et al., 2025). For context, both glufosinate and glyphosate are widely used non-selective herbicides, making the acquisition of such resistance to one or both herbicides particularly consequential. There is a similar observation from cancer circulomes where a core set of 27 eccDNA-driven genes were found across cancerous, non-cancerous diseased and healthy samples (Gu et al., 2025). Together, these data argue eccDNA should not be viewed as a passive byproduct of genomic instability, but as a regulated and versatile vehicle for genetic variability.

### 2.2 Functional and adaptive roles of eccDNA

The properties outlined above position eccDNAs as independent, functional genetic units – more analogous to bacterial plasmids than chromosomes – that can increase genomic and phenotypic flexibility without altering the chromosomal DNA. By acting as mobile reservoirs of repetitive DNA, coding sequences, and regulatory sequences, eccDNAs constitute a conserved adaptive architecture that can be generated, modified, and lost as conditions demand, a genomic “shock-absorber” in response to environmental change. eccDNAs generated “on the fly” can produce rapid, enduring, yet reversible evolutionary outcomes, enabling eukaryotes to explore adaptive phenotypic variation and providing a potential mechanism for resilience in fluctuating and stressful environments.

To understand how such rapid, reversible adaptation is encoded, we next consider the gene content and structure of eccDNAs across taxa. We begin by noting that large eccDNAs in many plants, including blackgrass (*Alopecurus myosuroides*), *Amaranthus* species, rice, and Arabidopsis, as well as in other eukaryotes such as yeast, mammals, and birds have been shown to contain full-length genes. These references are summarized in **Table 3** and their findings overturn earlier assumptions that eccDNAs were too small to harbor complete genes or that their primary function was mediated solely through small RNA pathways (Paulsen et al., 2019). eccDNA-encoded genes can be highly enriched within circulomes. In blackgrass, for example, up to 22% of sequenced eccDNAs are predicted to contain one or more genes, with an average of two genes per eccDNA and some eccDNAs carrying as many as 15 (Fu et al., 2023).

**Table 3:** Overview of species in which gene−containing eccDNAs have been identified.

Category/taxon	Species	Representative references	Notes
Plants –Weeds	*Alopecurus myosuroides* (blackgrass)	(Fu et al., 2023)	Abundant eccDNAs carrying herbicide-related genes.
	*Amaranthus* spp. (*A. palmeri*, *A. tuberculatus*, etc.)	(Koo et al., 2018; Molin et al., 2020a, 2020, 2020; Koo et al., 2022; Spier Camposano et al., 2022; Koo et al., 2023; Pereira and Dunning, 2023; Carvalho-Moore et al., 2025; Ni et al., 2025; Raiyemo et al., 2025; Song et al., 2025; Yao et al., 2025)	Megabase-scale eccDNA replicons containing full-length *EPSPS* and additional genes that are transcribed; key system for large eccDNA biology.
Plants –Crops	*Oryza sativa* (rice)	(Zhuang et al., 2024; Ni et al., 2025)	Large multi-fragment eccDNAs; some containing full genes.
Plants – Model Systems	*Arabidopsis thaliana*	(Cohen et al., 2008; Navrátilová et al., 2008; Wang et al., 2021; Merkulov et al., 2023; Zhang et al., 2023; Zaidi et al., 2025)	TE-derived and gene-containing eccDNAs reported across multiple studies.
Fungi	*Saccharomyces cerevisiae* and others	(Libuda and Winston, 2006; Gresham et al., 2010; Galeote et al., 2011; Møller et al., 2015b; Hull et al., 2019; Blount et al., 2023)	Numerous gene-bearing eccDNAs, including adaptive amplifications and stress-responsive circles.
Mammals	Various mammals	(Alt et al., 1978; Wu et al., 2019; Gu et al., 2025)	ecDNA often megabase-scale, carrying full oncogenes or multi-gene loci.
Birds	*Columba livia domestica* (pigeon)	(Møller et al., 2020)	Large eccDNAs observed that include full gene content.

In plant eccDNA research there is not a defined size cutoff that formally defines “small” versus “large” eccDNAs. Instead, literature typically reports the empirical size distributions their methods recover and use qualitative language such as microDNA, small circles, and massive replicon that reflects both biology and the measurement platform. Within the published literature, there appear to be two practical approaches. For example, most genome-wide surveys using Circle-seq/mobilome-style enrichment recover eccDNAs largely in the bp-to-low-kb range, while a smaller set of studies document rare but biologically important eccDNAs in the tens-to-hundreds of kilobases. For example, a rice Circle-seq study reports eccDNAs spanning 74 bp to 5,196 bp, with substantial enrichment in the ~200–400 bp “microDNA” range (Zhuang et al., 2024). In *Arabidopsis*, genome-wide profiling likewise emphasizes abundant short eccDNAs but also reports larger size modes, including peaks around ~219 bp, ~24.7 kb, and ~109 kb (Wang et al., 2021), and a study in Palmer amaranth reporting distributions around 6kb on average (Spier Camposano et al., 2022). These studies illustrate that plant eccDNA can extend well beyond a few kilobases even outside specialized resistance systems. By contrast, an eccDNA that is implicitly considered “large” is uniquely described as the eccDNA replicon (Koo et al., 2018; Molin et al., 2020c; Carvalho-Moore et al., 2025), which was captured and sequenced using bacterial artificial chromosomes (BACS) or HiFi sequencing chemistry by Pacific Biosciences. Given these published distributions, our proposed convention is to consider small eccDNAs as those <10 kb (capturing the dominant bp–kb populations in genome-wide surveys), an intermediate class of 10–100 kb (where some plant datasets show substantial circles), and large eccDNAs as >100 kb.

This shift in understanding has been made possible largely by advances in bioinformatic pipelines for eccDNA analysis (Mehta et al., 2020; Mann et al., 2022; Yuan et al., 2024) and by improvements in sequencing technologies, particularly long-read circulome sequencing (i.e. Ni et al. (2025) and Zaidi et al. (2025)) which circumvent the need to map short-read datasets onto an existing reference genome. Because eccDNA detection is method-sensitive with different enrichment strategies and computational pipelines recovering only partially overlapping eccDNA sets and producing method-dependent size spectra and genomic annotations (Gao et al., 2024), plant circulome studies should explicitly acknowledge that inferences about TE- versus gene-derived eccDNA abundance and size distributions may vary with protocol choice.

Interestingly, genes that frequently appear on eccDNA also tend to be highly expressed (Møller et al., 2015b; Shoura et al., 2017; Wu et al., 2019), suggesting a feedback relationship in which transcription promotes eccDNA formation, and gene amplification, which in turn can further elevate transcription. Transcriptomic analyses show that a subset of these protein-coding genes carried by eccDNAs are actively transcribed (Molin et al., 2020c; Ni et al., 2025), indicating that these sequences are not genomic debris but functional genetic elements. This coupling between transcriptional activity, eccDNA formation, and copy-number gain has clear functional consequences when the amplified genes encode adaptive traits. By amplifying beneficial genes and enhancing their expression, eccDNAs provide a rapid mechanism for adaptation. The best-characterized example is the amplification of *5-ENOLPYRUVYLSHIKIMATE-3-PHOSPHATE SYNTHASE* (*EPSPS*) amplification on eccDNA, which underlies glyphosate resistance in several plant species (Koo et al., 2018; Molin et al., 2020a, 2020; Koo et al., 2022; Spier Camposano et al., 2022; Carvalho-Moore et al., 2025). In *Amaranthus palmeri*, resistant plants harbor eccDNAs that carry multiple copies of *EPSPS* along with 58 additional genes associated with detoxification, replication, recombination, and transport (Molin et al., 2020c). Similar enrichment of key players in xenobiotic metabolism, such as glutathione S-transferases (GSTs), cytochrome P450s, and ABC transporters, has been observed in the circulomes of herbicide-resistant blackgrass (Fu et al., 2023). Functional domain-based analyses across multiple plant species consistently identify NADH dehydrogenases, leucine-rich repeat proteins, and DNA-binding domains on eccDNAs (Molin et al., 2020c; Spier Camposano et al., 2022; Fu et al., 2023), activities typically associated with stress signaling and response. In rice, eccDNA formation correlates with developmental timing and nutrient stress, and eccDNA-associated genes show differential expression under low nitrogen and phosphate conditions (Ni et al., 2025). Together, these observations indicate that eccDNA-mediated gene amplification and functional enrichment provide a mechanistic basis for rapid adaptation, linking genomic plasticity to stress responses and the evolution of resistance in plants.

**Figure 2:**
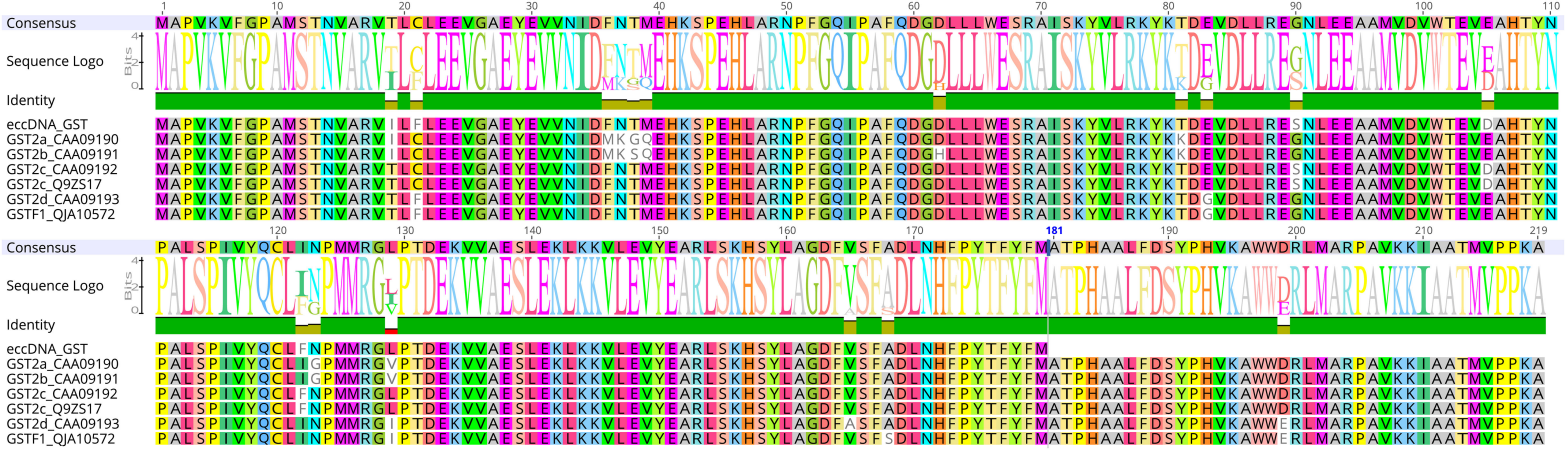
The eccDNA-encoded GSTF1 identified in Fu et al (Fu et al., 2023). is different from the GSF1 variants previously published and available on NCBI.

A key question in plant eccDNA biology is whether eccDNA-encoded coding sequences (eccCDSs) differ functionally from their chromosomal counterparts. Notably, circulomes from resistant blackgrass carry a variant of the plant-specific phi class of GST (AmGSTF1) - a gene associated with non-target-site herbicide resistance (Cummins et al., 2013; Franco-Ortega et al., 2021; Lowe et al., 2024). Interestingly, the eccDNA-encoded AmGSTF1 is predicted be 39 amino acids shorter than its chromosomal homolog (Fu et al., 2023) (**Figure 2**). Although further research is needed to determine whether this variant is expressed and/or translated, its presence in resistant blackgrass circulomes raises the possibility that eccCDSs may be structurally, and therefore functionally, distinct from other alleles. Such structural divergence suggests that eccDNAs may not only increase gene dosage but also generate novel protein variants with altered enzymatic properties.

Because eccDNA originates from genomic DNA yet evolves independently, it represents a parallel source of genetic innovation. eccCDSs variants carried on eccDNA can diverge from their chromosomal templates without compromising core genome integrity, allowing even individual cells to explore new allelic or regulatory states. As described above, eccDNAs carrying single or multiple copies of advantageous eccCDSs may spread through tissues and across generations (Koo et al., 2018) and between species (Koo et al., 2023; Zhang et al., 2024), thereby expanding the genetic landscape accessible to populations. Collectively, these features position eccDNA as a rapid-response system for genomic innovation. By reshuffling, amplifying, and mobilizing genetic material, eccDNA provides stressed plants with a flexible mechanism to generate new functional diversity and adapt to environmental challenges.

Moreover, focal amplification of chromosomal elements onto eccDNA can alter their regulatory context, often releasing genes and/or transposable elements from chromosomal constraints. In chromosomes, DNA is tightly packaged with histones and marked by epigenetic modifications that create regions of variable accessibility (Candela-Ferre et al., 2024). By contrast, eccDNA within the circulome lacks the higher-order compaction of chromosomes, at least in cancer cells, making it more accessible and readily transcribed (Wu et al., 2019). Consistent with this, impairing DNA methylation alters eccDNA profiles (Zaidi et al., 2025), indicating that epigenetic state influences eccDNA composition and abundance. Thus, DNA (e.g. a gene or transposons, which are discussed further below) that is highly repressed on the chromosome may become highly active once incorporated into eccDNA. In this way, the circulome can modulate gene expression and therefore protein production from eccCDSs. eccCDSs with altered chromatin accessibility (Wu et al., 2019), can achieve dramatically increased copy numbers (Alt et al., 1978; Kuttler and Mai, 2007; Koo et al., 2018; Molin et al., 2020c; Koo et al., 2022) and display distinct transcriptional (Paulsen et al., 2019; Wu et al., 2019; Molin et al., 2020c; Ni et al., 2025) and methylation (Zaidi et al., 2025) profiles compared to their chromosomal counterparts. Together, these changes can directly affect cellular function and stress responses. For a comprehensive discussion of these concepts in the context of cancer progression, drug resistance, and viral infection, see Yao et al. (2025) or Wu et al. (2019).

### 2.3 Repetitive elements and the circulome

Repetitive elements comprise a substantial yet variable proportion of the circulomes studied to date. The Fu et al. (2023) dataset shows that TE-derived sequences are a major and recurring component of blackgrass eccDNA, with Ty3-retrotransposons LTR elements particularly prominent among annotated domains. Similar observations have been made in other plants (e.g. Cohen et al. (2008); Kwolek et al. (2022), and Molin et al. (2020c)) and other species (e.g. Møller et al. (2015b) and Shoura et al. (2017)). Supporting this, recent long-read eccDNA atlas in Arabidopsis revealed that a substantial fraction of eccDNAs maps to TE loci, both full-length and truncated, spanning diverse TE superfamilies (Merkulov et al., 2023; Zaidi et al., 2025). These findings collectively suggest that TE sequences contribute significantly to the composition of plant eccDNA.

Repetitive elements are normally subject to tight genomic regulation through multiple mechanisms, including epigenetic repression (DNA methylation and histone modification), RNA interference (RNAi), repeat-binding protein complexes, and genome stability systems such as those acting at telomeres (Wallrath et al., 2022). However, when TE sequences are excised into eccDNAs, they become decoupled from chromosomal constraints, allowing them to potentially escape local silencing, replicate autonomously, or evolve distinct regulatory profiles. This dynamic interplay implies that eccDNA could serve as a catalyst for TE-driven genomic innovation, especially under conditions where epigenetic control is relaxed or destabilized. As we are starting to recognize the role of repetitive element movement in genome evolution (e.g. Gisby and Catoni (2022)), it may be important to consider the positioning of eccDNA in this process.

Transposon regulation has long been associated with environmental stress responses (Wallrath et al., 2022), and this relationship extends to eccDNA dynamics. Stress may both promote TE movement into eccDNA and enhance TE mobility or amplification once eccDNA forms. For example, under combined heat and epigenetic stress, ONSEN (an LTR/Copia retrotransposon in Arabidopsis) generates eccDNA, but only when these stressors are applied together, highlighting the stimulus-specific and context-dependent nature of eccDNA formation (Merkulov et al., 2023). Moreover, eccDNAs derived from LTR/Copia, Ty3-retrotransposons, and Helitron elements vary across tissues, developmental stages, and epigenetic mutant backgrounds (Zaidi et al., 2025), further linking TE mobilization, methylation state, and eccDNA biogenesis. With these functional and repetitive-element components in view, we next consider how eccDNA’s autonomy and modularity could be leveraged or disrupted in plant biotechnology.

### 2.4 Taking a circulome-based view of plant biotechnology

In bacterial systems, plasmids revolutionized molecular biology by providing autonomous, transferable genetic elements capable of driving gene expression independently of the host genome (Helinski, 2022). These circular DNA molecules enabled rapid trait acquisition, horizontal gene transfer, and programmable genetic manipulation. If properly understood and harnessed, eccDNA may offer a parallel system for plants – one that is native, versatile, and potentially transformative.

Plant eccDNAs share several key features with bacterial plasmids (**Figure 1**):

- Autonomous replication: eccDNAs contain autonomously replicating sequences (ARS) that allow them to propagate independently of chromosomal DNA (Cohen et al., 2006; Cohen and Lavi, 2009; Molin et al., 2020b; Yang et al., 2023; Zhang et al., 2024).
- Stable yet stochastic inheritance: eccDNAs are inherited through both somatic and germline pathways, including pollen-mediated and graft-transmissible transfers, enabling non-Mendelian transmission of traits (Koo et al., 2018, 2023; Zhang et al., 2024).
- Modular architecture: eccDNAs can carry complete gene coding sequences along with regulatory elements such as promoters and terminators, forming self-contained expression units (Guo et al., 2023; Zhong et al., 2023; Ni et al., 2025).

These properties suggest that, if properly understood, eccDNA could conceptionally function in a manner analogous to plant plasmids, with the potential to drive gene expression in a context-specific and stress-responsive manner. Unlike cis- or transgenes that are integrated into the genome, eccDNA-based systems may avoid epigenetic silencing and positional effects, offering a more flexible and potentially reversible route to trait manipulation. The concept of programmable plants that carry synthetic- or naturally-enriched circular DNA designed to express specific traits therefore represents an intriguing possibility for plant biotechnology. An improved understanding of which coding and regulatory regions might contribute to eccDNA functionality may, in the future, offer insights into how eccDNA-based constructs could be designed to reflect native resilience processes. In principle, such constructs could be transiently expressed in somatic tissues or stably inherited through non-Mendelian pathways, offering a novel route to crop improvement without direct modification of chromosomal DNA. Importantly, this approach aligns with the growing demand for non-GMO solutions in agriculture. eccDNA-based systems could enable trait enhancement without genome editing, potentially bypassing regulatory hurdles and public resistance associated with transgenic crops.

In considering eccDNA-enabled trait engineering, two complementary routes emerge that include a discovery/selection and design/selection. A discovery/selection strategy leverages low-dose, recurrent abiotic selection (e.g., repeated heat pulses, drought–recovery cycles, salinity escalation, oxidative challenge, nutrient limitation) at a population-scale to enrich individuals that sustain performance with limited fitness cost. Conceptually, this can be intensified by intermittent epigenetic “relaxation” (transient chromatin opening and/or reduced methylation pressure) to raise the probability of focal amplification and eccDNA formation, thereby expanding the substrate on which selection can act. Prior plant studies support the plausibility of this approach where stress combined with epigenetic perturbation has been shown to increase extrachromosomal retrotransposon DNA and mobilization outputs, and long-read eccDNA profiling under epigenetic stress has revealed dramatic, family-specific bursts of eccDNA production (Thieme et al., 2017; Merkulov et al., 2023, 2024). After each selection cycle, eccDNA profiling could be performed to reveal enriched circles and/or repeatedly amplified genomic loci that co-segregate with improved stress performance.

Conversely, a design-first route starts from trait biology (QTL/GWAS, stress-responsive transcriptomics) and translates those targets into eccDNA payloads built with context-dependent regulatory logic (ideally stress-inducible and tissue-specific rather than constitutive). Early-stage experiments as transient episomes provides rapid functional performance in somatic tissues or cell-based systems, while later iterations can incorporate maintenance features inspired by naturally persistent plant eccDNA systems. Importantly, the strongest path forward is likely a hybrid situation where selection identifies what biology can amplify and tolerate, while engineering refactors those enriched regions into a minimal, programmable eccDNA unit with predictable expression and controllable persistence.

Conceptually, these plant-focused opportunities are strengthened by developments in other systems showing that circular DNA can be both evolutionarily consequential and experimentally tractable. For example, circular-DNA intermediates have been proposed as a mechanism that can reshuffle genomic architecture across generations, potentially contributing to measurable changes in gene order in mammals (Holt et al., 2023). In oncology, ecDNA is now being treated as an actionable therapeutic substrate, with first-in-human clinical studies explicitly framed around ecDNA-directed strategies (e.g., CHK1 inhibition with BBI-355; NCT05827614 as per ClinicalTrials.gov (2025)). Parallel advances in CRISPR-enabled ecDNA mapping/enrichment and emerging CRISPR-based ecDNA elimination approaches further demonstrate that extrachromosomal DNA can be targeted as a distinct molecular entity (Hung et al., 2022).

One holy grail of crop biotechnology engineering is to ensure plants are climate resilient so they can survive the abiotic challenges of tomorrow’s agricultural ecosystems. Ensuring that our crops are phenotypically plastic, defined as the ability of an organism to alter its physiology, morphology, or development in response to environmental stimuli, may be key to this. In agricultural weeds, phenotypic plasticity is not merely a nuisance but a superpower. Weeds such as blackgrass have demonstrable capacity to rapidly respond to herbicides (Comont et al., 2020), waterlogging (Harrison et al., 2024), and to use the experience of one stress to respond in a way that allows them to survive subsequent stresses (Mohammad et al., 2022). While traditional models attribute such adaptability to chromosomal mutations (Kersten et al., 2023), poly-genic traits (Cai et al., 2023), or epigenetic regulation (Chen et al., 2023), the evidence provided throughout this review shows the circulome also plays a pivotal role in expanding the phenotypic repertoire of plants, especially in weeds (Molin et al., 2020b, 2020; Spier Camposano et al., 2022; Fu et al., 2023). As explained above, unlike the genome, the circulome is less constrained; eccDNAs can replicate autonomously and be stochastically yet stably inherited, and those that work are kept, or even transferred between individuals (Koo et al., 2023; Zhang et al., 2024). These properties position the circulome as the unique vehicle that could drive rapid genomic innovation in future-proofed plants. Harnessing this system is important and harnessing it from plants with demonstrated capacity to rapidly adapt to changing climate and agricultural practices is clever. Weedy circulomes could transform our ability to engineer resilience, adaptability, and stress tolerance in crops; the very weeds that challenge conventional agriculture could be the key to unlock the agricultural innovations needed for tomorrow’s sustainable agriculture.

Translating insights from weedy circulomes face multiple challenges. Firstly, recovering intact circles is difficult because eccDNAs are fragile and heterogeneous. Getting these circles into plant cells is then the second challenge. Standard transformation systems (via *Agrobacterium* or biolistics) typically accommodate inserts under ~50 kb, far smaller than many functional eccDNAs (e.g., the ~400kb kb *EPSPS* replicon). New delivery platforms that are optimized for large DNAs, such as synthetic minichromosomes or nanoparticle-based systems, could overcome this barrier. Once in, species-specific differences in eccDNA biogenesis and maintenance, which are often tied to transposons, recombination, and epigenetic control, might mean that circles that persist in weeds may be unstable or rapidly lost if compatible replication or maintenance features are missing. Heritability is another constraint. EccDNAs frequently segregate in a non-Mendelian fashion producing mosaic expression, cell to cell variability, and potential loss across generations, especially in polyploid or outcrossing species. Regulatory and biosafety considerations are also factors. As mentioned above, the probability for horizontal movement of eccDNA within and between species may require extensive environmental risk assessment. Finally, even when constructs work in controlled settings, field performance may be unpredictable. Stress-responsive eccDNA effects can be highly context dependent, making multi-site, multi-year validation essential to ensure durable efficacy without unintended ecological impacts.

### 2.5 EccDNA as stress sensing, dosage tuning elements

Current evidence suggests that eccDNAs behave as emergent “stress sensing” elements of the plant cell whose formation, composition, and persistence are tightly coupled to environmental and developmental cues. Building on details explained above, their copy number and sequence content shift dynamically in response to xenobiotic exposure, nutrient limitation, and combined abiotic or epigenetic stress, with specific circles reproducibly enriched under defined conditions. In *Amaranthus palmeri*, for example, the *EPSPS* eccDNA replicon expands under glyphosate selection and can acquire additional herbicide target genes, yielding dual glyphosate/glufosinate resistance (Carvalho-Moore et al., 2025). In blackgrass, circulomes from resistant populations are enriched for detoxification genes central to xenobiotic metabolism (Fu et al., 2023). TE-derived eccDNAs arise only under precise combinations of heat and epigenetic stress, highlighting the stimulus-linked nature of eccDNA biogenesis (Merkulov et al., 2023). Nutrient stress applied to rice reshapes the eccDNA pool, with circles carrying low-N and low-P responsive genes that show altered expression under deficiency (Ni et al., 2025). Together with yeast and cancer data showing transcription coupled eccDNA formation at highly expressed loci and rapid dosage shifts under selection, these observations argue that eccDNA formation is an interesting route by which stressed cells explore increased gene copy number and novel regulatory contexts without committing to irreversible chromosomal rearrangements (Arrey et al., 2022).

Seen through this lens, eccDNA mediated gene amplification represents a conditionally directed response: stress exposure does not necessarily target amplification at specific loci, but biases eccDNA formation and retention toward genomic regions that are both transcriptionally active and functionally beneficial under the prevailing conditions. The resulting gene- and TE-rich eccDNA can achieve very high copy numbers, experience relaxed chromatin constraints, and segregate unevenly, generating a spectrum of cellular phenotypes on which selection can act within a single generation. This stress-coupled modulation of eccDNA dosage suggests that plant circulomes function as dynamic, non-Mendelian reservoirs of stress-responsive alleles. When stress is applied, eccDNA copy number and diversity increase; when stress is relieved, costly circles are recombined into other structures, lost, or diluted. In agricultural weeds, this mechanism may underpin the remarkable plasticity with which populations respond not only to herbicides but also to waterlogging, drought, and other abiotic pressures, complementing polygenic and epigenetic routes to adaptation (Pereira and Dunning, 2023).

### 2.6 Manipulating eccDNA to control the tempo of adaptive evolution

If eccDNA is indeed a central component within the cell through which plants modulate gene dosage and explore adaptive phenotypes under stress, then deliberately manipulating eccDNA formation, content, and inheritance offers a conceptual route to controlling the speed of adaptive evolution. On the acceleration side, one can envision designing synthetic eccDNA “resilience modules” inspired by weed circulomes. These modules would couple stress inducible promoters and autonomously replicating sequences to suites of genes involved in drought tolerance, heat resilience, nutrient acquisition, or xenobiotic detoxification. Under defined abiotic stress exposures (e.g., episodic heat waves, chronic low N or P, or exposure to novel chemistries), such circles could amplify and segregate through somatic and germline lineages, rapidly elevating the dosage of protective pathways without permanent chromosomal modification. Because eccDNA can also be lost when selection is relaxed, engineered circulomes might provide a reversible “adaptive overlay” that can be modulated up or down by adjusting environmental conditions or chemical inducers, which may be attractive for coping with fluctuating weather patterns.

Conversely, the same logic suggests strategies to slow evolution in pests, pathogens, and weeds. In *Amaranthus* and blackgrass, targeting eccDNA maintenance through inhibition of replicon encoded replication or recombination factors, disruption of tethering motifs, or epigenetic re-silencing of TE-rich circles could reduce the rate at which resistant genotypes arise and are maintained under herbicide pressure. Lessons from oncology, where ecDNA directed therapies (e.g., CHK1 inhibitors by ClinicalTrials.gov (2025)) and CRISPR-based approaches to isolate or selectively degrade ecDNA (Hung et al., 2022) are entering preclinical and clinical testing, indicate that extrachromosomal DNA can be targeted as a distinct molecular entity. Analogous concepts could be translated to plant pathogens whose effector or fungicide resistance loci are carried on eccDNA, or to transgenic traits like Bt, where limiting the eccDNA-mediated spread or increased copy number of resistance alleles could prolong durability. Realizing these possibilities will require (i) high resolution maps of eccDNA content and dynamics in both crops and associated biota across stress gradients, (ii) mechanistic dissection of plant eccDNA replication, segregation, and degradation pathways, and (iii) development of tools to modulate circulome behavior without unacceptable off target effects.

## 3 Discussion

We have painted eccDNA as a dynamic, stress-responsive layer of genomic plasticity that complements chromosomal DNA and enables rapid, non-Mendelian adaptation. Evidence across plant systems shows that eccDNAs amplify stress-related genes, modulate regulatory contexts, and mobilize repetitive elements, collectively expanding phenotypic flexibility without permanent genomic alteration. These properties position eccDNA as both an important mechanism for adaptive resilience and a potential biotechnological tool. Moving forward, key priorities include defining the “minimal functional unit” of plant eccDNA, elucidating mechanisms governing its formation and inheritance, and developing strategies to harness or inhibit eccDNA for crop improvement and resistance management. By leveraging insights from weeds and integrating advances in circulome mapping, plant biotechnology can explore eccDNA as a native, programmable system for climate-ready agriculture.
